# A systematic review and meta-analysis on the differentiation of glioma grade and mutational status by use of perfusion-based magnetic resonance imaging

**DOI:** 10.1186/s13244-022-01230-7

**Published:** 2022-06-07

**Authors:** Lusien van Santwijk, Valentina Kouwenberg, Frederick Meijer, Marion Smits, Dylan Henssen

**Affiliations:** 1 Department of Medical Imaging, Radboud University Medical Center, Geert Grooteplein Zuid 10, 6525 EZ Nijmegen, The Netherlands; 2 Department of Radiology and Nuclear Medicine, Erasmus MC, University Medical Center Rotterdam, Rotterdam, The Netherlands

**Keywords:** Dynamic contrast enhancement magnetic resonance perfusion imaging, Dynamic susceptibility contrast magnetic resonance perfusion imaging, Glioma, Molecular classification

## Abstract

**Background:**

Molecular characterization plays a crucial role in glioma classification which impacts treatment strategy and patient outcome. Dynamic susceptibility contrast (DSC) and dynamic contrast enhanced (DCE) perfusion imaging have been suggested as methods to help characterize glioma in a non-invasive fashion. This study set out to review and meta-analyze the evidence on the accuracy of DSC and/or DCE perfusion MRI in predicting IDH genotype and 1p/19q integrity status.

**Methods:**

After systematic literature search on Medline, EMBASE, Web of Science and the Cochrane Library, a qualitative meta-synthesis and quantitative meta-analysis were conducted. Meta-analysis was carried out on aggregated AUC data for different perfusion metrics.

**Results:**

Of 680 papers, twelve were included for the qualitative meta-synthesis, totaling 1384 patients. It was observed that CBV, ktrans, Ve and Vp values were, in general, significantly higher in IDH wildtype compared to IDH mutated glioma. Meta-analysis comprising of five papers (totaling 316 patients) showed that the AUC of CBV, ktrans, Ve and Vp were 0.85 (95%-CI 0.75–0.93), 0.81 (95%-CI 0.74–0.89), 0.84 (95%-CI 0.71–0.97) and 0.76 (95%-CI 0.61–0.90), respectively. No conclusive data on the prediction of 1p/19q integrity was available from these studies.

**Conclusions:**

Future research should aim to predict 1p/19q integrity based on perfusion MRI data. Additionally, correlations with other clinically relevant outcomes should be further investigated, including patient stratification for treatment and overall survival.

**Supplementary Information:**

The online version contains supplementary material available at 10.1186/s13244-022-01230-7.

## Key points

- Perfusion MR imaging shows a promising method to characterize glioma non-invasively.
- Significant higher perfusion metrics are observed in IDH-wildtype glioma.
- The effects of 1p/19q mutations on perfusion metrics are understudied and remain unelucidated.

## Introduction

Following the 2016 World Health Organization (WHO) classification system of tumors of the central nervous system, the high-grade glioma group can be divided in two subgroups. One subgroup comprises the anaplastic oligodendroglioma IDH mutant and 1p/19q codeleted, and the anaplastic oligodendroglioma not otherwise specified. The second subgroup comprises the IDH mutant glioblastoma, the IDH wildtype glioblastoma, and the glioblastoma not otherwise specified [1]. Knowledge on the exact mutational status of glioma is not only important for classification, it also has significant impact on prognosis [2] and treatment strategy [3–5]. With regard to low grade gliomas, two groups of gliomas can be distinguished. The first groups consist of oligodendroglial tumors which are isocitrate dehydrogenase (IDH) mutant and 1p/19q codeleted The second groups consist of astrocytic tumors. It is comprised of (1) IDH mutated, 1p/19q non-codeleted diffuse astrocytoma, (2) the IDH wildtype astrocytoma, and (3) the diffuse astrocytoma not otherwise specified [1].

However, the recently published WHO 2021 classification system has placed even more emphasis on the molecular characteristics of glioma subtypes. The group of diffuse astrocytic and oligodendroglial gliomas can be subdivided based on the IDH mutations. IDH wildtype tumors are classified as high-grade gliomas, without exception. In order to be classified as glioblastoma (IDH wildtype; grade 4), nuclear ATRX loss has to be present. Additionally, IDH wild-type diffuse astrocytic tumors in adults without the histological features of glioblastoma, but with one or more of three genetic parameters (telomerase reverse transcriptase gene [TERT] promoter mutation, epidermal growth factor receptor [EGFR] gene amplification, or combined gain of entire chromosome 7 and loss of entire chromosome 10 [+ 7/ − 10]) are now also classified as glioblastoma. In the 2021 classification, all *IDH*-mutant diffuse astrocytic tumors with intact 1p/19q chromosomes are considered a single type called astrocytoma, *IDH*-mutant with WHO grades ranging from 2 to 4. Grading of these tumors takes into account molecular findings such as the homozygous deletion of CDKN2A/B, which is associated with a worse prognosis. *IDH*-mutant astrocytomas with these molecular alterations will be classified as WHO grade of 4, even if microvascular proliferation or necrosis is absent [6]. Additionally, IDH mutant oligodendroglial gliomas with codeleted 1p/19q chromosomes are considered oligodendrogliomas. While the establishment of the sophisticated molecular markers to classify gliomas is an important advance in glioma diagnosis, all of the literature which is covered within this review is based on the 2016 WHO classification of central nervous system tumors [6, 7].

To this end, artificial intelligence applied to conventional MRI sequences (i.e., pre- and post-contrast T1-weighted, T2-weighted and T2-weighted FLAIR images) to predict mutational status has provided promising results in recent years (for a review, see [8]). In addition, various signs have been identified which can help the radiologist to predict the molecular status of glioma in the daily clinical setting. For example, the T2-FLAIR mismatch sign has been found to be a reliable non-invasive marker for identification of IDH mutant astrocytomas [9].

Bearing in mind the pathophysiological differences between various glioma subtypes and the related changes in the gliomas vasculature, perfusion-based imaging could increase the diagnostic accuracy of non-invasive characterization of glioma subtypes. For example, oligodendroglial tumors are characterized by a branching pattern of vascularization, whereas astrocytic glioma shows a distinctively different vascularization [10]. Therefore, perfusion based MR imaging (either dynamic susceptibility contrast (DSC) or dynamic contrast enhancement (DCE) perfusion MR imaging) has been the subject of research to non-invasively identify molecular characteristics [11, 12].

DSC-perfusion MR imaging relies on the susceptibility induced signal loss on T2*-weighted sequences, resulting from a bolus of gadolinium-based contrast agent passing through the capillaries. The most commonly used DSC perfusion parameter is Cerebral Blood Volume (CBV). CBV can be estimated by use of the area under the curve (AUC) of the signal intensity-time curve [13, 14]. However, more recent studies compute CBV maps by integrating the transverse relaxivity changes which occur dynamically over a first-pass injection followed by leakage correction due to the leaky blood–brain barrier in most tumors (for a recent overview and recommendations, see [15]). DCE-perfusion MR imaging relies on the evaluation of T1 shortening induced by a gadolinium-based contrast agent bolus leaking from the blood vessels into the tissue. Pharmacokinetic modeling can be used to derive various values including, Ve and Vp. ktrans represents the capillary permeability; Ve represents the fractional volume of the gadolinium-based contrast agent in the extravascular-extracellular space; Vp represents the fractional volume of the of the gadolinium-based contrast agent in the plasma space [13].

Although various studies with different methodologies and outcomes have been published since the release of the WHO 2016 classification system of glioma, a comprehensive overview of the accuracy of perfusion based MR imaging to predict the molecular characteristics of glioma is still lacking. In addition, a systematic overview of the literature on this topic could help to shape future research and daily clinical practice to focus on the most promising technique (either DSC- or DCE-perfusion MRI). The aim of the current paper was therefore to provide an overview of the relevant literature with regard to the use of DSC and DCE perfusion imaging used to differentiate glioma grade and mutational status.

## Materials and methods

### Search strategy and inclusion/exclusion methodology

This systematic review and meta-analysis was conducted following the Preferred Reporting Items for Systematic Reviews and Meta-Analyses (PRISMA) statement [16]. Databases searched for literature were: Medline (accessed through PubMed), EMBASE, Web of Science, and the Cochrane Library. The full search strategies for each database are made available in the Additional file 1. Cross-referencing was used to add relevant literature to the database. Searches were conducted between May 1, 2020 and January 1, 2021. Inclusion criteria were: (1) the use of either DSC or DCE perfusion MRI; (2) the inclusion of patients suffering from glioma; (3) glioma grading and classification by use of the WHO 2016 classification system [1]; and (4) the aim of the study needed to comprise the non-invasive classification of histopathological features and/or molecular characteristics (WHO grade, IDH genotype and/or 1p/19q codeletion status). Besides, papers needed to report results as quantitative measures (e.g., sensitivity, specificity, mean accuracy and/or mean area under the receiver operator curve (AUC)). Papers were excluded if they were based on animals or non-human samples or a pediatric population. Letters, preprints, case reports, congress proceedings, and narrative reviews were excluded as well.

All papers were independently assessed by two researchers in three steps. First, screening on title and abstract was carried out. Second, full-text analysis was employed to assess whether the papers met the inclusion- and/or exclusion criteria. Finally, information was extracted from the included papers. Researchers met periodically to discuss their findings and resolve discrepancies. Standardized tables were used to acquire the information of interest from the included articles by two researchers (LvS and DH) independently. Data extracted from each study were (a) first author and year of publication, (b) number of patients included, (c) mean age of the included participants, (d) gender of the included participants, (e) use of DSC and/or DCE, (f) which histopathological/molecular outcome was assessed, (g) perfusion based MR imaging metrics and (h) accompanying statistics (e.g., AUC value, standard deviation, 95% confidence interval (CI) and/or standard error). Performance was expressed in accuracy, AUC and/or sensitivity and specificity for each outcome. Extracted data were cross-checked afterward, and discrepancies were resolved in consensus.

### Qualitative meta-synthesis and quantitative meta-analysis

Eligible literature was synthesized qualitatively following the PICO-strategy as proposed by Eriksen et al. [17]. Also, quality of primary diagnostic accuracy studies was assessed using the QUADAS-2. Meta-analysis was conducted on the AUC and the 95% Confidence Interval (95%-CI) using a random effects model. From the included studies, perfusion metrics and the aforementioned statistics were extracted. If one of these variables was missing, the researchers aimed to re-calculate the value when possible [18]. In addition, corresponding authors were contacted to provide missing details, with up to two reminders send by e-mail. When not all necessary data could be acquired, studies could not be meta-analyzed. Meta-analyses were conducted on different subgroups of target conditions. Meta-analysis was performed with the use of OpenMetaAnalyst (MetaAnalyst, Tufts Medical Center) [19] and/or SPSS (version 25; IBM Corp., Armonk, NY) and results were displayed in forest plots. The Higgins test was used to test for heterogeneity between included studies. Low heterogeneity between groups is marked with an *I*^2^ < 40%, whereas considerable heterogeneity is indicated by *I*^2^ > 75% [18].

## Results

**Fig. 1:**
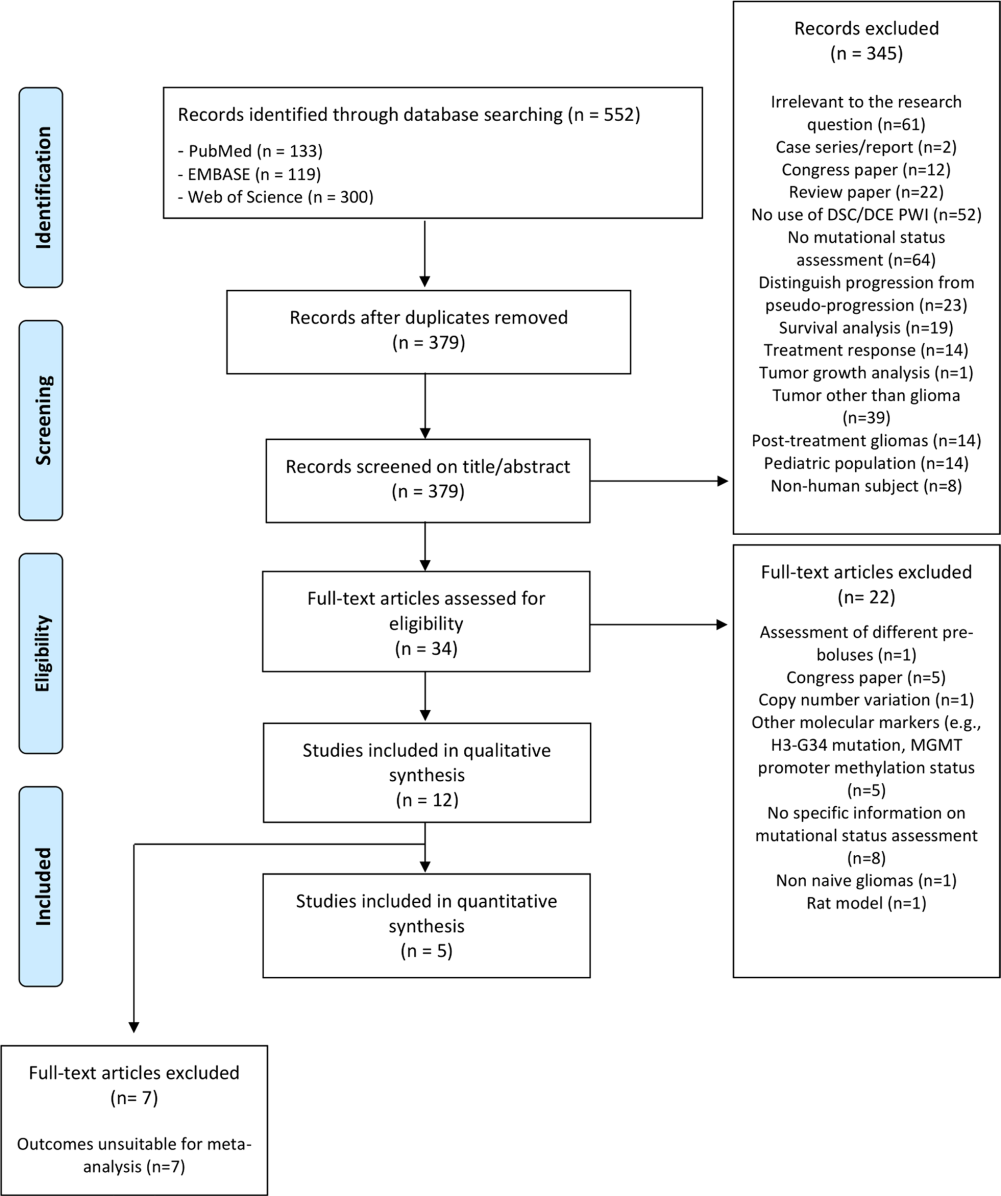
PRISMA flow diagram

A total of 552 studies were identified after systematic searching. Duplicates were removed and 379 papers were systematically screened on title and abstract resulting in the inclusion of 34 papers for full-text analysis. Reasons for exclusion of the 345 papers are provided in Fig. 1. After full-text analysis, the investigators met to discuss the identified non-consensus papers to resolve disagreements and to reach consensus. Of the 34 papers, 12 could be included in the qualitative meta-synthesis. Twenty-two papers were therefore excluded (details provided in Fig. 1). No discrepancies between the judgement of the two researchers remained after discussion, resulting in the final inclusion of 12 papers for the qualitative meta-synthesis [20–31] (Fig. 1). Five papers provided sufficient data to be included in the quantitative meta-analysis [22, 24, 26, 27, 29] (Table 1).

**Table 1:** Overview of the included studies

Authors (year)	*N*	Age (years)	*M*/*F*	MRI perfusion method + details on analysis	Glioma types and grades included	Outcome assessed	Major findings
Brendle et al. (2020) [30]	56	Mean age 48.0 ± 16.0	33/23	DSC Gradient echo sequence Pre-bolus of contrast agent was applied (0.025 mmol/kg gadobutrol) Mean rCBV values BSW-model based leakage correction	WHO grade II: 29 WHO grade III: 20 WHO grade IV: 7 IDHmut: 32 IDHwt: 24	IDH mutation status & 1p/19q codeletion status	The mean rCBV was significantly different between the astrocytic tumors, oligodendrogliomas and IDHmut astrocytic tumors and oligodendrogliomas and IDHwt astrocytic tumors
Choi et al. (2019) [20]	463	Mean age 52.2 ± 14.8	272/191	DSC Gradient echo sequence Pre-bolus of 0.1 mmol/kg gadobutrol Mean rCBV values No information on postprocessing with regard to leakage-correction	WHO grade II: 32 WHO grade III: 142 WHO grade IV: 289 IDHmut: 328 IDHwt: 125 1p/19q codel: 56 1p/19q non-codel: 407	IDH mutation status	The IDH mutation status predictions had an accuracy, sensitivity, and specificity of 92.8%, 92.6%, and 93.1%, respectively, in the validation set with an AUC of 0.9 (95%-CI 0.969–0.991). In the test set, the IDH genotype prediction had an accuracy, sensitivity, and specificity of 91.7%, 92.1%, and 91.5%, respectively, with an AUC of 0.95 (95%-CI 0.898–0.982)
Hempel et al. (2019) [21]	100	Mean age 51.4 ± 14.7	55/45	DSC Gradient echo sequence Pre-bolus of 0.1 mmol/kg gadobutrol Mean rCBV values* BSW-model based leakage correction	WHO grade II: 40 WHO grade III: 30 WHO grade IV: 30 IDHmut: 31 IDHwt: 46 1p/19q codel: 23	IDH mutation status	rCBV was significantly lower in patients with IDHmut than in those with the IDHwt. Mean rCBV values showed a sensitivity/specificity of 52/91 for the prediction of IDH mutation status with an AUC of 0.780
Hilario et al. (2019) [22] **(X)**	49	Range 16–78	28/21	DSC Gradient echo sequence Pre-bolus of 0.1 mmol/kg gadobutrol No rCBV values provided BSW-model based leakage correction	LGG: 8 HGG: 41 IDHmut: 10 IDHwt: 31	IDH mutation status	Significant differences in the values of leakage (*p* = 0.01), Ktrans (*p* = 0.002), Vp (*p* = 0.032) and Ve (*p* < 0.001) between high-grade and low-grade diffuse gliomas were observed The highest AUC was demonstrated by the DCE permeability parameters Ktrans (AUC = 0.838, CI95% 0.710–0.967, *p* = 0.003) and Ve (AUC = 0.878, CI95% 0.768–0.988, *p* = 0.001). Among IDHmut and IDHwt highgrade gliomas, there were significant differences in leakage (*p* = 0.004) and Ktrans values (*p* = 0.028) showing lower leakage and Ktrans values
				DCE Dynamic gradient echo sequence 5 mL of gadobutrol at a rate of 3 mL/s Mean Ktrans, Vp, Ve and Kep values Model based leakage correction			
Lee et al. (2018) [23]	39	Mean age 43.6 (range 21–82)	19/20	DSC Gradient echo sequence No prebolus administration described Mean rCBV values* BSW-model based leakage correction	WHO grade II: 19 WHO grade III: 20	WHO grade	Ktrans, Kep, and Ve showed tendencies toward higher values in oligodendroglial tumors than astrocytic tumors
				DCE Dynamic gradient echo sequence 0.1 mmol/kg gadobutrol at a rate of 4 mL/s Mean Ktrans Model based leakage correction			
Lee et al. (2020) [24] *(X)*	110	IDHmut.- 1p/19q noncodel: mean age 40.7 ± 12.8; IDHwt: mean age 51.2 ± 14.0; IDHmut-1p/19q codel: mean age 46.5 ± 11.7	56/54	DSC Gradient echo sequence Pre-bolus of 0.1 mmol/kg gadoterate meglumine Mean normalized rCBV values* BSW-model based leakage correction	WHO grade II: 45 WHO grade III: 65 IDHmut: 65 IDHwt: 45 1p/19q codel: 46 1p/19q non-codel: 19	IDH mutation status & 1p/19q codeletion status	When using nCBV skewness, the AUC was found to be 0.690 (95%-CI: 0.573, 0.807) with a sensitivity of 84.2 and specificity of 59.3 to distinguish IDHmut-1p/19q noncodel from the other two groups
Sudre et al. (2020) [25]	333	Mean age 48.9 (range 20–81)	198/135	DSC No details provided on imaging protocol and whether or not a prebolus was administered Mean rCBV values* BSW-model based leakage correction	WHO grade II: 101 WHO grade III: 74 WHO grade IV: 158 IDHmut: 151 IDHwt: 182	WHO grade & IDH mutation status	Shape, distribution and texture features showed significant differences across mutation status. WHO grade II-III differentiation was mostly driven by shape features, while texture and intensity feature were more relevant for the III-IV separation. Increased number of features became significant when differentiating grades further apart from one another. Gliomas were correctly stratified by mutation status in 71% and by grade in 53% of the cases
Wang et al. (2020) [31] **(X)**	30	IDHmut: mean age 42.8 (range 22–67) IDHwt: mean age 47.9 (range 19–78)	17/13	DCE Dynamic gradient echo sequence Pre-bolus of 0.1 mmol/kg gadopentetate dimeglumine at a rate of 4 mL/s Mean Ktrans, Vp, Ve Model based leakage correction	WHO grade II: 22 WHO grade III: 8 IDHmut: 18 IDHwt: 12	IDH mutation status	Compared to IDHmut LGGs, IDHwt LGGs exhibited significantly higher perfusion metrics (*p* < 0.05)
Wu et al. (2020) [26]	44	63.8 ± 7.4	27/17	DSC Gradient echo sequence No pre-bolus administration** Mean rCBV values Gamma-variate curve fitting leakage correction	WHO grade III: 19 WHO grade IV: 25 IDHmut: 19 IDHwt: 25 1p/19q codel: 7 1p/19q non-codel: 3	IDH mutation status	Compared with IDHwt, IDHmut had significantly decreased rCBV at the high-angiogenic enhancing tumor habitats and low-angiogenic enhancing tumor habitats
Xing et al. (2017) [27] *(X)*	42	IDHmut: mean age 35.8 ± 9.1 IDHwt: mean age 46.0 ± 18.4	26/16	DSC Gradient echo sequence Pre-bolus of 0.1 mmol/kg gadobenate dimeglumine Mean rCBVmax values* BSW-model based leakage correction	WHO grade II: 24 WHO grade III: 18 IDHmut: 17 IDHwt: 25	IDH mutation status	The threshold value of < 2.35 for relative maximum CBV in the prediction of IDH mutation status provided a sensitivity, specificity, positive predictive value, and negative predictive value of 100.0%, 60.9%, 85.6%, and 100.0%, respectively
Xing et al. (2019) [28]	75	IDHmut: mean age 52.2 ± 12.7 IDHwt: mean age 40.7 ± 10.8	41/34	DSC Gradient echo sequence Pre-bolus of 0.1 mmol/kg gadobenate dimeglumine Mean rCBVmax values* BSW-model based leakage correction	WHO grade IV: 75 IDHmut: 10 IDHwt: 65	IDH mutation status	Both rCBVmax-t and rCBVmax-p showed significant differences between IDHmut and IDHwt. The optimal cutoff values in prediction of IDH-m. < 7.27 for rCBVmax-tumor, and < 0.97 for rCBVmax-peri-enhancing region
Zhang et al. (2020) [29] *(X)* **(X)**	43	47.0 ± 13.0	20/23	DSC Gradient echo sequence DSC imaging followed DCE imaging; no separate pre-bolus was administered Mean rCBVmax values* BSW-model based leakage correction	WHO grade II: 14 WHO grade III: 14 WHO grade IV: 15 IDHmut: 20 IDHwt: 23	IDH mutation status	Ve (AUC = 0.816, sensitivity = 0.84, specificity = 0.79) and Kep (AUC = 0.818, sensitivity = 0.76, specificity = 0.78) provided the highest differential efficiency for IDH mutation status prediction
				DCE Dynamic gradient echo sequence Pre-bolus of 0.1 mmol/kg gadodiamide Mean Ktrans, Vp, Ve* Model based leakage correction			

Marked in italics are the publications included in the meta-analysis on the use of DSC-value; Marked in bold are the publications included in the meta-analysis on the use of the DCE-values

AUC, area under the curve; DCE, dynamic contrast enhancement magnetic resonance perfusion imaging; DSC, dynamic susceptibility contrast magnetic resonance perfusion imaging; F, females; HGG, high-grade glioma; IDH, isocitrate dehydrogenase; IDHmut, mutation of the isocitrate dehydrogenase gene(s); IDHwt, wild-type isocitrate dehydrogenase gene(s); Kep, rate constant between the extravascular extracellular space and blood plasma; ktrans, volume transfer coefficient; LGG, low grade glioma; M, males; MRI, magnetic resonance imaging; nCBV, normalized cerebral blood volume; rCBV, relative cerebral blood volume; rCBVmax-t, maximum relative cerebral blood volume in the tumor-enhancing region; rCBVmax-p, maximum relative cerebral blood volume in the peri-enhancing region; Ve, fractional volume of the extravascular extracellular space; Vp, fractional blood plasma volume; WHO, World Health Organization; 95%-CI, 95%-confidence interval

*Study provides a variety of perfusion statistics (either DSC or DCE metrics; values included mean, standard deviation and a variety of percentiles)

**Lack of pre-bolus administration was compensated by use of a flip-angle of 60° which reduced T1 effects [44]

Using the QUADAS2 (QUality Assessment tool for Diagnostic Accuracy Studies), the most current version of the QUADAS tool of the QUADAS task force, the risk of bias was considered low in all included studies (Table 2).

**Table 2:** Combined effect size for the different DCE/DSC parameters

	Ktrans	Ve	Vp	CBV
Effect size	0.813	0.844	0.777	0.832
Standard error	0.02	0.03	0.03	0.03
95%-CI lower limit	0.726	0.766	0.683	0.749
95%-CI upper limit	0.900	0.921	0.871	0.914

ktrans, volume transfer coefficient; rCBV, relative cerebral blood volume; Ve, fractional volume of the extravascular extracellular space; Vp, fractional blood plasma volume; 95%-CI, 95%-confidence interval

### Qualitative meta-synthesis

The twelve included studies [20–31] totaled 1384 patients (792 males; 592 females) suffering from glioma. Gliomas could be subdivided into WHO grade II (*n* = 326); WHO grade III (*n* = 410) and WHO grade IV (*n* = 599). Regarding the IDH genotype, 701 gliomas were IDH-mutated and 603 tumors expressed an IDH wildtype genotype. 1p/19q codeletion (WHO 2021 Oligodendroglioma WHO grade 2 or 3) was observed in 132 tumors; non-codeletion of 1p/19q chromosome arms was observed in 429 tumors. All included papers used histopathological/molecular assessment by a trained neuropathologist who adhered to the WHO 2016 glioma classification as the gold standard.

Eight papers used DSC perfusion MRI [20, 21, 24–28, 30]; three papers used both DCE and DSC perfusion MRI [22, 23, 29]; one paper used DCE perfusion MRI only [26]. Two papers used artificial intelligence methods to assess different perfusion metrics between various subtypes of gliomas [20, 25], whereas the other publications used more traditional statistics.

As assessed by DSC perfusion MRI, IDHmut glioma displayed significantly lower rCBV values as compared to IDHwt glioma [21, 26–28, 30]. When using a retrospectively determined rCBVmax threshold value of < 2.35, the authors described a sensitivity/specificity of 100%/61% and AUC of 0.82 (95%-CI: 0.66–0.93) when differentiating IDHmut (either WHO 2021 Astroctytoma grade 2, 3 or 4 or WHO 2021 Oligodendroglioma grade 2 or 3) and IDHwt gliomas [27]. By use of the skewness of normalized CBV (nCBV) values (normalized by use of the CBV value of the normal-appearing contralateral centrum semiovale), IDHmut, 1p/19q non-codeleted glioma (WHO 2021 Astrocytoma grade 2, 3 or 4) could be distinguished from IDHwt glioma and IDHmut, 1p/19q codeleted glioma (WHO 2021 Oligodendroglioma) with a sensitivity/specificity of 84%/59% (AUC-value of 0.690 and 95%-CI 0.573–0.807). IDHmut, 1p/19q non-codeleted gliomas (WHO 2021 Astrocytoma grade 2, 3 or 4) showed significant lower nCBV values compared to the IDHmut, 1p/19q codeleted gliomas (WHO 2021 Oligodendroglioma grade 2 or 3) and the IDH wildtype gliomas [24].

When using DCE perfusion imaging, IDHmut HGG (either WHO 2021 Astroctytoma grade 3 or 4 or WHO 2021 Oligodendroglioma grade 3) showed significantly lower ktrans values as compared to IDHwt HGG (WHO 2021 Astrocytoma grade 4) [22]. In oligodendroglial tumors (WHO 2021 Oligodendroglioma, IDHmut, 1p/19q-codeleted; Grade 2 or 3), however, Lee et al. found that ktrans, Kep and Ve showed tendencies toward higher values as compared to astrocytic tumors [23]. Ve and Vp values were found to be significantly lower in IDHmut glioma (WHO 2021 Astrocytoma and WHO 2021 Oligodendroglioma) as compared to IDHwt glioma, regardless of WHO II-IV grading [22, 29]. Based on Ve and Kep values, a sensitivity/specificity of 84%/79% and 76%/78% was observed with regard to differentiate IDH mutation status [29]. The study of Hilario et al. also suggested that ktrans, Vp and Ve could be used to differentiate between LGG and HGG non-invasively [22].

Studies using artificial intelligence showed promising results with regard to prediction of IDH mutation status. Choi et al. showed that a convolutional long short-term memory model with an attention mechanism had an accuracy, sensitivity, and specificity of 92.8%, 92.6%, and 93.1%, respectively, in the validation set (AUC: 0.98; 95%-CI 0.969–0.991) with regard to IDH genotype prediction by use of DSC perfusion MRI. In the test set, an accuracy, sensitivity, and specificity of 91.7%, 92.1%, and 91.5% were observed, respectively. The AUC value of the IDH genotype prediction demonstrated to be 0.95 with a 95% CI ranging between 0.898 and 0.982. Subsequent analysis of the signal intensity curves of DSC imaging elucidated high attention on the combination of the end of the pre-contrast baseline, the up/downslopes of signal drops, and/or post-bolus plateaus for the curves used to predict IDH genotype [20]. Another study showed that when using a random forest algorithm, shape, distribution and rCBV-extracted features elucidated significant differences across mutation status. WHO grade II-III differentiation was mostly driven by shape features, while texture and intensity feature were more relevant for the distinguishing of III and IV. Based on this random forest algorithm, gliomas were correctly stratified by mutation status in 71% and by grade in 53% of the cases [25].

### Meta-analysis

**Fig. 2:**
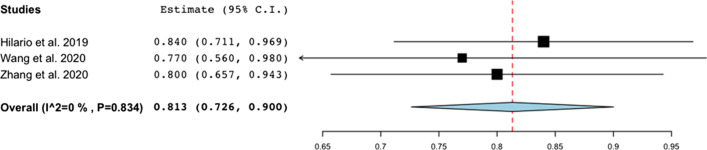
Forest-plot of the area under the curve (AUC) of the receiver operator curve (ROC) of the different perfusion metrics in predicting IDH mutation status. IDH, isocitrate dehydrogenase, ktrans, volume transfer coefficient; rCBV, relative cerebral blood volume; Ve, fractional volume of the extravascular extracellular space; Vp, fractional blood plasma volume; 95%-CI, 95%-confidence interval

Meta-analysis of the data (*n* = 237 patients) showed that CBV values have an accuracy of correctly predicting IDH genotype with an AUC of 0.832 and a standard error of 0.03 (95%-CI 0.75–0.91). When using DCE parameters (*n* = 122), an AUC of 0.81, 0.84 and 0.78 is observed for ktrans, Ve and Vp, respectively. Standard errors (and 95%-CI) for ktrans, Ve and Vp were found to be 0.02 (95%-CI 0.73–0.90), 0.03 (95%-CI 0.77–0.92) and 0.03 (95%-CI 0.68–0.87), respectively (Table 2). The corresponding Forest-Plots of the different perfusion metrics are provided in Fig. 2. *I*^2^ analysis showed that included DCE-MRI studies were homogeneous (*I*^2^ < 1%). In the individual analyses of ktrans, Ve and Vp, studies were found to be non-significantly heterogeneous (*p* = 0.834; *p* = 0.548; *p* = 0.519, respectively). The meta-analysis of DSC-MRI studies showed to have moderate heterogeneity (*I*^2^ = 35%; *p* = 0.215). The role of perfusion MRI metrics in predicting the 1p/19q-codeletion status could not be meta-analyzed using the acquired data.

## Discussion

This systematic review and meta-analysis shows that perfusion MRI can be used to effectively predict IDH genotype non-invasively following the WHO 2016/2021 glioma classification. Different DSC or DCE perfusion parameters were found to have an equal performance regarding the non-invasive prediction of IDH genotype. Prediction of the 1p/19q-codeletion status could not be meta-analyzed using the acquired data.

The role of perfusion MRI in non-invasive glioma classification can be significant and can be explained by the different glioma vasculature fingerprints which provide a specialized microenvironment for glioma cells [32]. Within HGG, blood vessels are abnormal and display a distinct gene expression signature which differs from the genotype of blood vessels in normal brain tissue [33–35]. These genotypic differences result in high expression of certain angiogenic factors, including vascular endothelial growth factor, transforming growth factor β2, and pleiotrophin [35–38]. In LGG, on the other hand, potential molecular alterations regarding angiogenesis have been investigated less extensively. In 2018, Zhang et al. demonstrated that WHO grade II glioma expressed an intermediate stage of vascular abnormality, less severe than that of glioblastoma vessels but distinct from normal vessels. Enhanced expression of laminin subunit alpha 4 and angiopoietin 2 in WHO grade II glioma was confirmed by staining of human tumor tissue microarrays. More specifically, IDHwt gliomas displayed a specific angiogenic gene expression signature (i.e., upregulation of Angiopoietin 2 and serpin family H) which resulted in enhanced endothelial cell migration and matrix remodeling. In the same study, transcription factor analysis indicated increased transforming growth factor beta and hypoxia signaling in IDHwt gliomas [39]. Based on these studies, we can conclude that gliomas with different IDH genotype have distinct molecular vascularization. In addition, the blood vessels in LGG displayed alterations in gene expression which partially overlapped with changes previously identified in HGG vessels [39]. As IDHwt glioma vessels have a distinct vascular gene expression pattern associated with vascular remodeling, these microstructural changes can be used to explain why IDHwt glioma show significantly higher perfusion metrics compared to IDHmut glioma. These insights in genotype and phenotype justify the use of perfusion MRI to predict IDH genotype. The role of 1p/19q codeletion status on angiogenesis and vascular growth, however, remains partially elusive. Previous research demonstrated that 1p/19q codeletion was associated with higher CBV values compared with glioma with intact alleles [40]. Another paper reported specific genotypic differences in oligodendroglioma by use of DSC perfusion MRI with significantly higher rCBVmax values in LGG with 1p/19q codeletion [41]. It is believed that 1p/19q codeleted LGG show an increased metabolism and angiogenesis and have an extensive internal vascular network. This is supported by the study of Kapoor et al. in which a significantly higher rCBVmax was observed in 1p/19q codeleted LGG (WHO 2021 Oligodendroglioma WHO grade 2). Additionally, an increased vascular endothelial growth factor expression, CD31, and CD105, was observed as compared with glioma with intact alleles [42].

The clinical usability of MRI perfusion imaging to predict IDH genotype remains partially elusive as the differences were based on aggregated results. Although several papers provided specific threshold values [27, 28], no clinically useful threshold values are available. The current review did not include studies arterial spin labeling (ASL) as a perfusion MR imaging method as only sparse literature with regard to ASL was found in exploratory literature searches. A recent paper by Wang et al. (2019) reported that only a mild correlation was found between the IDH1 genotypes and ASL derived glioma perfusion parameters. There was no significant association between 1p/19q codeletion and perfusion in grade II and III gliomas [43].

### Strength and limitations

By adhering to the 2016 WHO glioma classification to be included, some valuable papers needed to be excluded, though also resulted in rather homogeneous dataset to be meta-analyzed. One of the strengths of this reviews concerns the relative homogeneous imaging protocols which were meta-analyzed. For example, all DSC-imaging protocols were imaged after administering a pre-bolus injection of a gadolinium-based contrast agent. Also, for the included studies which investigated the diagnostic accuracy of DCE-imaging, mean perfusion values (i.e., Ve, Vp and ktrans). However, different studies used different values of perfusion parameters (mean rCBV vs. mean rCBV max values), which partially limits the generalizability of results [15]. The homogeneity of the meta-analyzed patients and histopathological outcomes (i.e., IDH genotype and 1p/19q codeletion status) strengthen the here described findings. Another limitation of the here applied methodology concerns the fact that this systematic review was executed without registration in an international database.

## Conclusion

This review and meta-analysis showed that accuracy of DSC parameters was not different from the accuracy of DCE parameters to non-invasive predict the IDH genotype in glioma patients. The use of perfusion MRI with regard to predicting 1p/19q codeletion status could not be determined using these data.

## Supplementary Information

**Additional file 1:** Search strategies.

## Data Availability

Data analyzed in this study will be made available upon reasonable request by contacting the corresponding author.

## Abbreviations

95%-CI: 95%-Confidence interval
AUC: Area under the curve
DCE MRI: Dynamic contrast enhancement magnetic resonance perfusion imaging
DSC MRI: Dynamic susceptibility contrast magnetic resonance perfusion imaging
HGG: High-grade glioma
IDH: Isocitrate dehydrogenase
IDHmut: Mutation of the isocitrate dehydrogenase gene(s)
IDHwt: Wild-type isocitrate dehydrogenase gene(s)
Kep: Rate constant of gadolinium efflux between the extravascular extracellular space and blood plasma
ktrans: Volume transfer constant
LGG: Low grade glioma
MRI: Magnetic resonance imaging
nCBV: Normalized cerebral blood volume
rCBV: Relative cerebral blood volume
rCBVmax-p: Maximum relative cerebral blood volume in the peri-enhancing region
rCBVmax-t: Maximum relative cerebral blood volume in the tumor-enhancing region
Ve: Fractional volume of the extravascular extracellular space
Vp: Fractional blood plasma volume
WHO: World Health Organization
