# Supplementary material for: A systematic review and meta-analysis on the differentiation of glioma grade and mutational status by use of perfusion-based magnetic resonance imaging
Source: Insights Imaging. 2022 Jun 7;13:102. doi: 10.1186/s13244-022-01230-7 (PMC9174367; doi:10.1186/s13244-022-01230-7)
Supplement: Supplementary file 1 — Additional file 1: Search strategies. [file 13244_2022_1230_MOESM1_ESM.docx]

**ELECTRONIC SUPPLEMENTARY MATERIAL**

**A systematic review and meta-analysis on the differentiation of glioma grade and mutational status by use of perfusion-based magnetic resonance imaging**

PUBMED

((glioma[MeSH Terms]) OR (gliomas[MeSH Terms])) AND ((DCE) OR (DCE MRI) OR (DCE-MRI) OR (Dynamic contrast enhanced) OR (Dynamic contrast-enhanced)) AND ((classification) OR (grading))

((glioma[MeSH Terms]) OR (gliomas[MeSH Terms])) AND ((DSC) OR (DSC MRI) OR (DSC-MRI) OR (Dynamic Susceptibility Contrast) OR (Dynamic-Susceptibility Contrast) OR (Dynamic Susceptibility-Contrast)) AND ((classification) OR (grading))

Web of Science

((glioma(tiab)) AND ((DCE(tiab)) OR (DCE MRI(tiab)) OR (DCE-MRI(tiab)) OR (Dynamic contrast enhanced(tiab)) OR (Dynamic contrast-enhanced(tiab))) AND ((classification(tiab)) OR (grading(tiab)))

((glioma(tiab)) OR (gliomas(tiab)) AND ((DSC(tiab)) OR (DSC MRI(tiab)) OR (DSC-MRI(tiab)) OR (Dynamic Susceptibility Contrast(tiab)) OR (Dynamic-Susceptibility Contrast(tiab)) OR (Dynamic Susceptibility-Contrast(tiab))) AND ((classification(tiab)) OR (grading(tiab)))

EMBASE

((glioma(ti,ab,kw)) AND ((DCE(ti,ab,kw)) OR (DCE MRI(ti,ab,kw)) OR (DCE-MRI(ti,ab,kw)) OR (Dynamic contrast enhanced(ti,ab,kw)) OR (Dynamic contrast-enhanced(ti,ab,kw))) AND ((classification(ti,ab,kw)) OR (grading(ti,ab,kw)))

((glioma(ti,ab,kw)) OR (gliomas(ti,ab,kw))) AND ((DSC(ti,ab,kw)) OR (DSC MRI(ti,ab,kw)) OR (DSC-MRI(ti,ab,kw)) OR (Dynamic Susceptibility Contrast(ti,ab,kw)) OR (Dynamic-Susceptibility Contrast(ti,ab,kw) OR (Dynamic Susceptibility-Contrast(ti,ab,kw))) AND ((classification(ti,ab,kw)) OR (grading(ti,ab,kw)))

The Cochrane Library

glioma in Title Abstract Keyword AND "dynamic susceptibility contrast" in Title Abstract Keyword AND "dynamic contrast enhanced magnetic resonance imaging" in Title Abstract Keyword AND "classification" in Title Abstract Keyword - (Word variations have been searched)

glioma in Title Abstract Keyword AND "dynamic susceptibility contrast enhanced magnetic resonance imaging" in Title Abstract Keyword AND "dynamic contrast-enhanced magnetic resonance imaging" in Title Abstract Keyword AND "classification" in Title Abstract Keyword - (Word variations have been searched)
